# Sea anemone *Bartholomea annulata* venom inhibits voltage-gated Na^+^ channels and activates GABA_A_ receptors from mammals

**DOI:** 10.1038/s41598-022-09339-x

**Published:** 2022-03-30

**Authors:** Antònia Colom-Casasnovas, Edith Garay, Abraham Cisneros-Mejorado, Manuel B. Aguilar, Fernando Lazcano-Pérez, Rogelio O. Arellano, Judith Sánchez-Rodríguez

**Affiliations:** 1 Posgrado en Ciencias del Mar y Limnología, Universidad Nacional Autónoma de México, Circuito Exterior S/N, Ciudad Universitaria, C.P. 04510 Coyoacán, Mexico Mexico; 2 Unidad Académica de Sistemas Arrecifales Puerto Morelos, Instituto de Ciencias del Mar y Limnología, Universidad Nacional Autónoma de México, Prolongación Niños Héroes s/n, Domicilio Conocido, C.P. 77580 Puerto Morelos, Quintana Roo Mexico; 3 Departamento de Neurobiología Celular y Molecular, Instituto de Neurobiología, Universidad Nacional Autónoma de México, Boulevard Juriquilla 3001, 76230 Juriquilla, Querétaro, C.P Mexico

**Keywords:** Drug discovery, Pharmacology

## Abstract

Toxin production in nematocysts by Cnidaria phylum represents an important source of bioactive compounds. Using electrophysiology and, heterologous expression of mammalian ion channels in the *Xenopus* oocyte membrane, we identified two main effects produced by the sea anemone *Bartholomea annulata* venom. Nematocysts isolation and controlled discharge of their content, revealed that venom had potent effects on both voltage-dependent Na^+^ (Na_v_) channels and GABA type A channel receptors (GABA_A_R), two essential proteins in central nervous system signaling. Unlike many others sea anemone toxins, which slow the inactivation rate of Na_v_ channels, *B. annulata* venom potently inhibited the neuronal action potential and the Na^+^ currents generated by distinct Na_v_ channels opening, including human TTX-sensitive (hNa_v_1.6) and TTX-insensitive Na_v_ channels (hNa_v_1.5). A second effect of *B. annulata* venom was an agonistic action on GABA_A_R that activated distinct receptors conformed by either α1β2γ2, α3β2γ1 or, ρ1 homomeric receptors. Since GABA was detected in venom samples by ELISA assay at low nanomolar range, it was excluded that GABA from nematocysts directly activated the GABA_A_Rs. This revealed that substances in *B. annulata* nematocysts generated at least two potent and novel effects on mammalian ion channels that are crucial for nervous system signaling.

## Introduction

Cnidarians are a diverse group of aquatic invertebrate animals that include jellyfishes, corals, hydrozoans, and sea anemones, among some others^1^. Some cnidarians are considered highly venomous on humans (e.g. Refs.^2–4^). This characteristic is associated with the synthesis and assembly of microscopic and sophisticated bioweapons known as nematocysts^5–7^. These extraordinary structures, are essentially, capsules that contain venom and are endowed with penetrant spines that allow effective venom administration on demand. Nematocysts are produced in specialized cells named nematocytes^5,8^. The venom contains a mixture of compounds^9^ that includes proteinaceous (e.g., peptides, proteins, and enzymes) and non-proteinaceous (e.g., purines, quaternary ammonium compounds, biogenic amines, and betaines) substances^10,11^, with neurotoxic effects and cytolytic activity, among others^12–19^. Nevertheless, the complex cnidarian venomous apparatus is still not fully understood. Recent studies show that venom source is not restricted to nematocysts. For instance, distinct stinging-cell structures called cassiosomes have been identified in the Rhizotomies order (scyphomedusae *Cassiopea xamachana*)^20^, and sea anemones such as *Nematostella vectensis* or *Anthopleura elegantissima* have ectodermal gland cells that produce toxins^21^. Also, it has been shown that different toxins are expressed throughout the animal life span^22^, even more, different tissue types of a particular animal produce specific venom components^23^. The diversity of active substances contained in the nematocysts, and other cnidarian structures, encourages a constant search for novel, specific and potent toxins acting on vertebrate membrane proteins, especially from the nervous system. However, the number of species studied until now remains extremely low (about 4%^1^). In this search for toxins, two bottlenecks slow down new developments in the area; the first is the discovery of relevant effects and the identification of molecular targets of toxins, the second, is undoubtedly, the purification of toxins and determination of their chemical nature. In this study, we explore the toxicity of the sea anemone *Bartholomea annulata* venom and aim to determine some of its molecular targets.

*Bartholomea annulata* (Anthozoa:Actiniaria: Aiptasiidae: Metridiodea) is a common sea anemone that inhabits the Caribbean Sea^24–27^ and produces three different types of nematocysts: basitrichous isorhizas, microbasic p-mastigophores, and microbasic amastigophores^26,28,29^. It has also been reported that *B. annulata* venom has deleterious effects on crabs, fishes, and even mice, generating spasmodic convulsions, cardiac arrhythmia, paralysis, and death^29,30^. Nevertheless, the bioactivity of *B. annulata* venom, which might explain its potent lethality, has not been directly explored on nervous system membrane proteins, in fact, venoms from the Metridioidea superfamily have been studied only in a limited number of species, one of the most comprehensive studies shows effects of *Exaiptasia diaphana* toxins acting on ion channels expressed in cardiomyocytes^31,32^. In here, we analyzed electrophysiologically the effects of *B. annulata* venom on a library of membrane proteins (from rat brain) heterologously expressed in *Xenopus* oocytes through mRNA injection. Once possible novel targets were identified, the characterization of effects was refined using various preparations for their electrophysiological analysis. We demonstrate that *B. annulata* nematocysts contain substances that produce two effects observed for the first time in the actiniarian group: (1) It potently inhibits the current flux through voltage-dependent Na^+^ channels, both TTX-sensitive and TTX-insensitive channels, and (2) it acts as a GABAergic agonist activating GABA type A channel receptors (GABA_A_R). The importance of both effects, for the central nervous system functioning, strongly suggests that these substances are central in the lethality of *B. annulata* venom and indicates the production and delivery of novel toxins from sea anemones.

## Results

### Bioactivity

**Figure 1:**
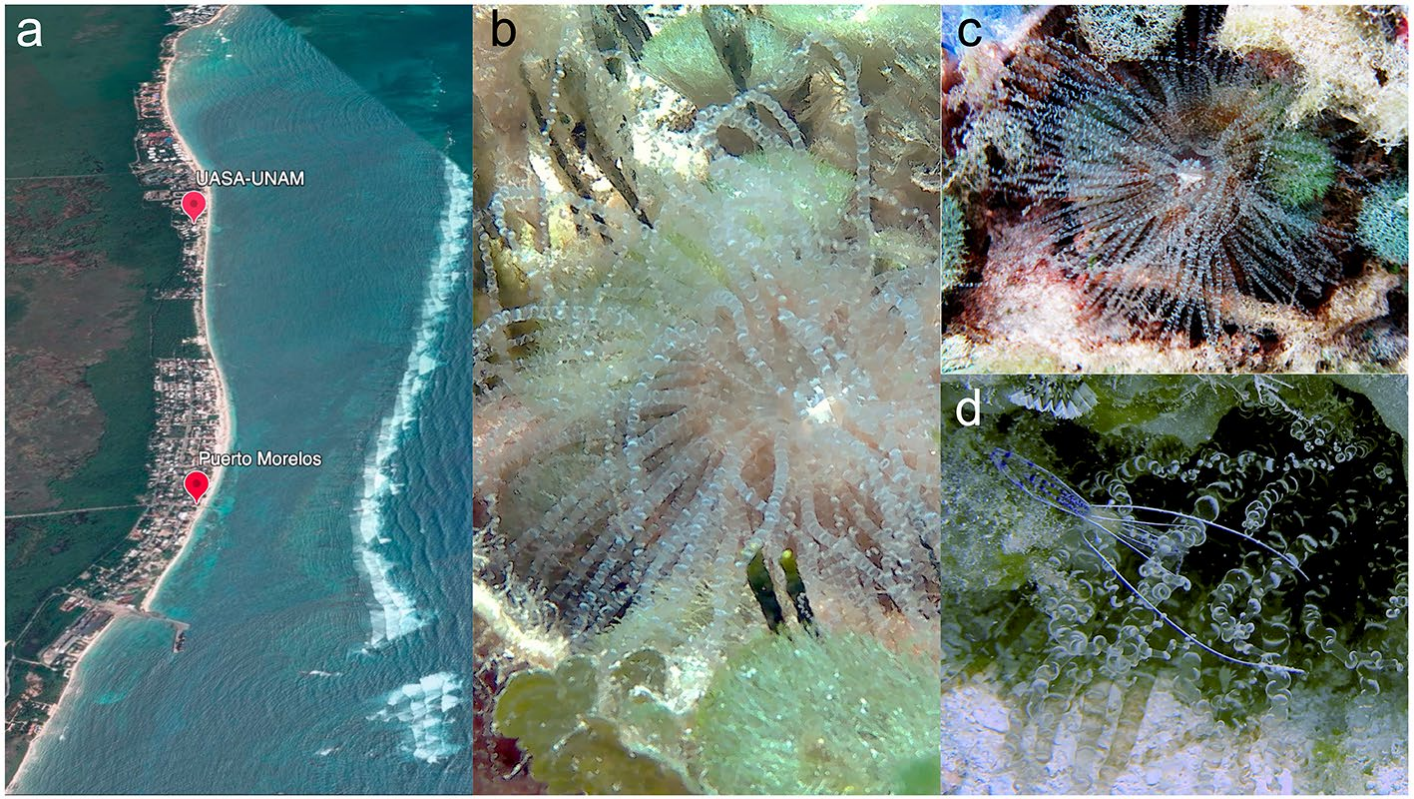
Sampling site location of *Bartholomea annulata* at the reef lagoon of Puerto Morelos, Quintana Roo. (**a**) Google Earth satellite image of Puerto Morelos Reef Lagoon, Quintana Roo, México; location points indicate Puerto Morelos town and the Unidad Académica de Sistemas Arrecifales UNAM (UASA-UNAM) (20° 50′ 45″ N 86° 52′ 08″ W and 20° 50′ 55″ N 86° 52′ 52″ W, respectively). (**b–d**) Sea anemone *Bartholomea annulata* is a member of family Aiptasiidae of the class Anthozoa; it is also commonly known as “corkscrew anemone”, due to spiral bands on its tentacles. Regularly is found inside crevices of rocks and coral rubble and has mutualist symbiosis with the cleaner shrimp *Ancylomenes pedersoni* (**d**) and the pistol shrimp *Alpheus armatus*.

To test bioactivity of *B. annulata* (Fig. 1) venom, either crude sea anemone extract (AE) or crude extract isolated from nematocysts (NE; see below) was administered to *Ocypode quadrata* crabs (n = 4 for each case) injecting 15–20 µg of the extract per g/crab in 50 µl PBS. In all cases, strong alterations, including tremor of legs, chelas, and eyes, uncontrolled movements, variable periods of freezing that increased until causing total paralysis, and finally death. Crabs (n = 4) injected with vehicle did not present any response. All these alterations were compatible with effects on the neuromuscular system and confirmed previous observations^29,33^.

**Figure 2:**
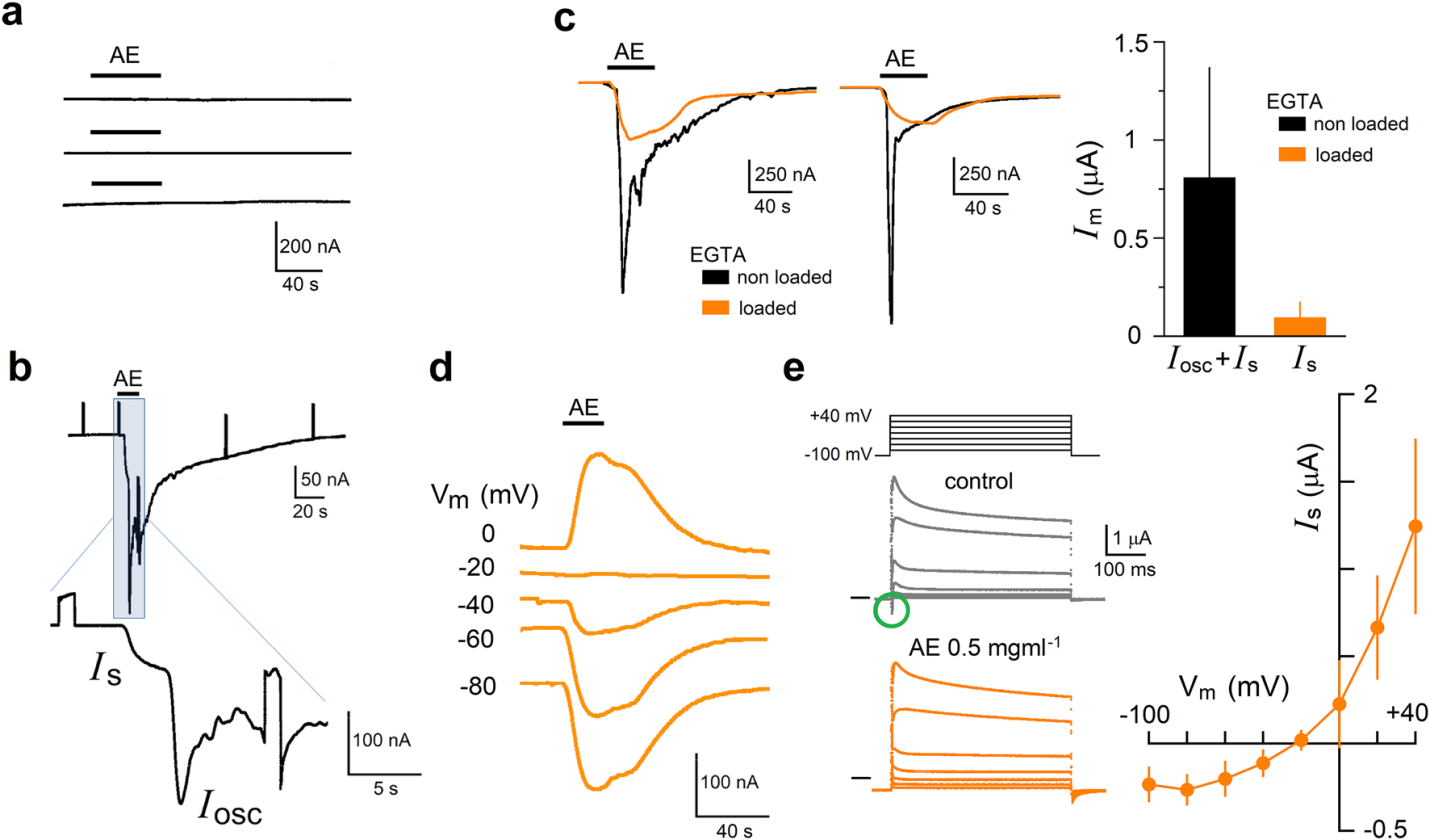
*Bartholomea annulata* venom produced three electrical responses in oocytes expressing membrane proteins from the rat brain. (**a**) Upper traces illustrate lack of current responses to application of anemone extract (AE, 0.1 mg ml^−1^) in three different control H_2_O-injected oocytes (3 frogs) under voltage-clamp held at − 60 mV, while in (**b**) traces show the current response elicited by AE in a mRNA-injected oocyte (expressing responses to different neurotransmitters as showed in Supplementary Fig. S1). Increase of the trace time-resolution signal in the insert (blue box) discloses activation of two currents with distinct temporal course, an early and smooth inward current, denoted *I*_s_, and a late and oscillatory inward current, denoted *I*_osc_. In (**c**) traces show that *I*_osc_ current observed in control response (black traces; EGTA non loaded oocytes) was eliminated in EGTA-loaded oocytes, while inward current EGTA-resistant corresponded with *I*_s_ time-course (orange traces). Total current responses (*I*_m_) obtained in 40 control and EGTA-loaded oocytes (right graph) indicated that *I*_s_ was of 93 ± 17.7 nA (mean ± S.D.). (**d**) *I*_s_ was elicited by 0.5 mg ml^−1^ AE superfusion in mRNA-injected oocytes that were EGTA-loaded, and holding the membrane potential as indicated in each trace, it is shown that *I*_s_ reverted close to − 20 mV, similar result was obtained in 2 more oocytes from different frog. (**e**) Traces illustrate a voltage-step protocol applied in EGTA-loaded oocytes (n = 4) held at − 100 mV in the absence of AE (control, gray traces) and during the application of the venom (0.5 mg ml^−1^; orange traces); the current–voltage relationship was built for the current elicited by AE subtracting the control current as it is shown in the graph. The black line to the left for each set of traces signals zero current. The green circle in control traces signals a fast inward current elicited at − 20 mV; this current, more probably due to opening of Na_v_ channels, was eliminated by AE application.

To conduct a broad screening of molecules involved in nerve transmission, we systematically analyzed the venom effect on membrane proteins from the rat brain that were heterologously expressed in *Xenopus* oocytes through mRNA injection^15,33,34^. It is well known that the oocyte has great experimental advantages for molecular target screening, including its capability to heterologously express functional proteins in its membrane that can be analyzed by electrophysiology. An example of the variety of electrical signals susceptible to analysis is shown in Supplementary Fig. S1. Using mRNA-injected oocytes, AE samples from *B. annulata* were tested. Figure 2 illustrates these experiments, first AE (0.1–1 mg ml^−1^) applied to control oocytes (H_2_O-injected; Fig. 2a) did not elicit any electrical response (n = 6, 3 frogs). However, AE application in oocytes expressing brain proteins generated several electrical responses (Fig. 2b–e), particularly three effects on three different responses were observed in all the oocytes tested. Thus, in oocytes held at − 60 mV, AE generated two types of currents that can be distinguished by their time-course (Fig. 2b) and their dependency on an intracellular Ca^2+^ concentration ([Ca^2+^]_i_) increase (Fig. 2c). These current responses are indicated as *I*_s_ (smooth) and *I*_osc_ (oscillatory) in Fig. 2b. The first response, *I*_s_, was an inward current associated with an increase in membrane conductance. The second response, *I*_osc_, began to increase a few seconds later and corresponded to an inward oscillatory current associated with an increase in conductance. Both responses, were co-activated while AE perfusion was maintained and were washed completely by NR superfusion. *I*_osc_ resembled the typical responses to various neurotransmitters when their receptors are expressed through brain mRNA injection in the oocyte (Fig. S1^35^). These are currents dependent on [Ca^2+^]_i_ increase promoted by phospholipase C activation and are due to Ca^2+^-dependent Cl^−^ channels opening^36,37^. To explore if *I*_osc_ elicited by the extract was activated through the same mechanism, oocytes that generated *I*_osc_ by AE, were washed, loaded with EGTA^38^, and then tested again with AE (Fig. 2c, orange traces). In all cases, AE elicited only *I*_s_ responses in EGTA-loaded oocytes, while *I*_osc_ were eliminated, indicating that *I*_s_ was not Ca^2+^-dependent, and confirming that *I*_osc_ was more probably activated through [Ca^2+^]_i_ increase. *I*_osc_ was the most robust response, whereas *I*_s_ showed lower amplitude, reaching values of 93 ± 83 nA (n = 22) (orange bar graph in Fig. 2c). No further analysis was performed on the mechanisms of *I*_osc_ activation. To explore the basic characteristics of *I*_s_, its reversal potential (E_rev_) was estimated in EGTA-loaded oocytes that were held at distinct membrane potentials while AE was superfused (Fig. 2d). *I*_s_ response had an E_rev_ close to − 20 mV in all cases tested (3 oocytes, 2 frogs). Also, Fig. 2e illustrates experiments where a protocol of pulses from − 100 to + 40 mV (in steps of 20 mV) was applied in the absence (control) and presence of AE. Thus, the *I*_s_ amplitude values elicited by AE in each potential were obtained by subtracting the corresponding control current values, and the results were plotted as illustrated. *I*_s_ presented an E_rev_ of − 22.7 ± 7.6 mV, like that obtained in Fig. 2d, suggesting a current mainly carried by Cl^−^ ions^36,39^. In the responses elicited by the pulse protocol, a fast voltage-dependent inward current that desensitizes within a few milliseconds was regularly activated (green circle in Fig. 2e; see also Supplementary Fig. S1). This current (denoted as *I*_Nav_) corresponds with the opening of voltage-dependent Na^+^ channels commonly expressed in brain mRNA-injected oocytes^40,41^. As depicted in Fig. 2e, *I*_Nav_ appears to be inhibited during the application of AE. Based on this initial screening, we decided to characterize in detail the AE effects on *I*_s_ activation and *I*_Nav_ inhibition.

### Inhibition of *I*_Nav_

**Figure 3:**
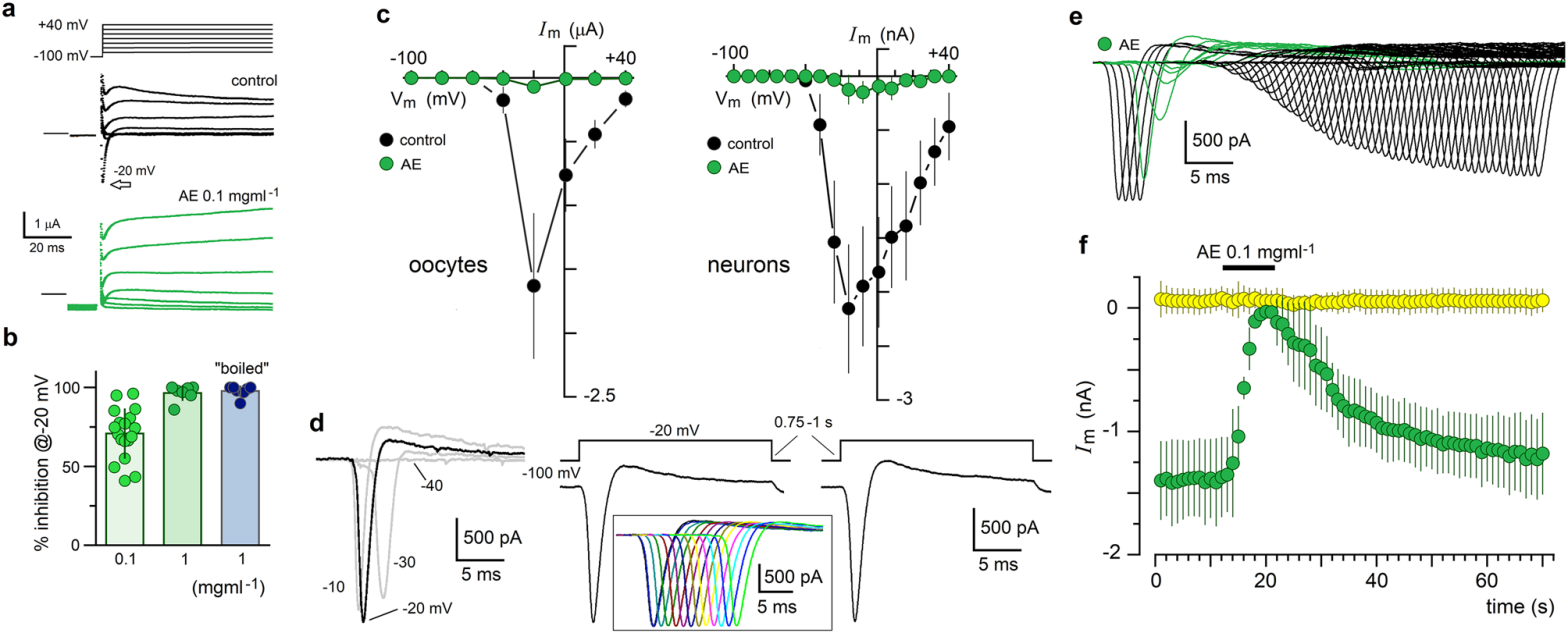
Anemone extract (AE) potently inhibited the *I*_Nav_ response in both mRNA-injected oocytes and in cultured cortical neurons. (**a**) Voltage-step protocol (from − 100 to + 40 mV) applied in mRNA-injected oocytes, held at − 100 mV, expressing voltage-dependent inward currents with a peak amplitude at − 20 mV, either in the absence (black traces) or the presence of AE (0.1 mg ml^−1^; green traces). (**b**) Voltage-dependent inward current at − 20 mV was potently inhibited by AE in concentrations of 0.1 mg ml^−1^ (18 oocytes, 7 frogs) and 1 mg ml^−1^ (7 oocytes, 3 frogs), the inhibitory effect was not eliminated in samples “boiled” by 45 min (7 oocytes, 3 frogs). (**c**) I–V relationship for the inward current response in oocytes (left graph; n = 8) suggested that it corresponds to opening of Na_v_ channels; this was confirmed recording Na_v_ currents in cultured cortical neurons (right I/V graph, black circles, n = 5) that were also potently inhibited by 1 mg ml^−1^ superfusion (green circles); in both cases currents were inhibited in the whole voltage-range analyzed. (**d**) Traces illustrate the *I*_Nav_ activated in neurons at − 30, − 20 and − 10 mV from a holding potential of − 100 mV. Maximal currents were regularly obtained at − 20 mV, to test the effect of extracts in this and subsequent figures, *I*_Nav_ was elicited periodically, stepping the membrane potential from − 100 to − 20 mV for 20 ms every 0.75–1 s as illustrated in the traces on the right. To show the complete time-course of a particular experiment, traces were superimposed as this is shown in the insert that illustrates 12 consecutive stimuli. (**e**) In neurons, *I*_Nav_ current was activated periodically as in (**d**). After monitoring the control amplitude (black traces), neurons were exposed to 0.1 mg ml^−1^ AE (green traces) for 10 s. During AE application *I*_Nav_ was rapidly inhibited, and after this, AE removal allowed current recovery. The graph in (**f**) shows the effect monitored in 5 neurons (mean ± S.D.) using the protocol in (**e**), top black bar signals AE application; inward currents (green circles) were eliminated while small outward currents depicted in yellow circles showed no change.

AE was applied to mRNA-injected oocytes expressing *I*_Nav_ to quantify its effect within a potential range of − 100 mV to + 40 mV and explore its reproducibility in different preparations. The peak *I*_Nav_ amplitude at − 20 mV was inhibited 96.5 ± 4.97% by 1 mg ml^−1^ AE (7 oocytes, 3 frogs, Fig. 3a–c). However, the extract at 0.1 mg ml^−1^ still caused a potent inhibition of 70.8 ± 15.89% (18 oocytes, 7 frogs). AE samples boiled for 45 min fully maintained their inhibitory activity (7 oocytes, 3 frogs), indicating that the inhibitory substance was thermostable (Fig. 3b).

*I*_Nav_ kinetics monitored in the oocyte, within the entire range of potentials tested, results uncertain due to the oocyte size. Due to this, and to explore the effect of AE on endogenous molecules (i.e., *I*_Nav_ in neurons), the following experiments were performed using cultured rat cortical neurons. Figure 3c shows the I–V relationships for *I*_Nav_ generated either in mRNA-injected oocytes or in neurons, and for both cases in the absence (black circles) and the presence (green circles) of AE; it was found that inhibition by AE was similar in both preparations. Thus, *I*_Nav_ inhibition occurred in the entire range of membrane potentials and, importantly, AE had the same effect on neurons; for example, 0.1 mg ml^−1^ AE inhibited neuronal *I*_Nav_ by 95.3 ± 3% (5 cells). Figure 3d illustrates a typical example of neuronal *I*_Nav_ elicited by 20 ms voltage steps within the − 10 to − 40 mV range from a holding potential of − 100 mV, regularly, the maximum current amplitude was obtained at − 20 mV. To study the time-course of the effects of AE, and other treatments, pulses to − 20 mV were repeated every 0.75–1 s for several seconds (70–85 s; Fig. 3d) in such a way that the different treatments were applied for 5–10 s after a control period, followed by washing with external solution. The insert in 3d illustrates a series of control responses obtained using this protocol that have been superimposed to show *I*_Nav_ reproducibility, the time scale corresponds to that of a single depolarizing pulse. Typical results testing the effect of different treatments using this protocol were illustrated with superimposed traces as it is shown in Fig. 3e, where the time-course *I*_Nav_ inhibition by AE was analyzed by depolarizing the neurons from − 100 mV as described, after 10–12 voltage steps in control external solution (black traces) AE was superfused for 5–10 s (green traces) and the effect on *I*_Nav_ was monitored. Finally, AE was washed and *I*_Nav_ recovery followed for several seconds (traces in black). As this is shown in the bottom graph, application of 0.1 mg ml^−1^ of AE produced *I*_Nav_ inhibition with a τ_50_ of 2.88 ± 0.03 s (Fig. 3f, green circles; 5 neurons), while smaller outward currents activated at this potential were not significantly affected (yellow circles; most likely corresponding to voltage-gated K^+^ channels). Recovery of inhibition required, in most cases, a 30–45 s wash. During *I*_Nav_ inhibition produced by AE, no change in the current inactivation rate was observed. For example, in a group of neurons (n = 6) the control time constant of inactivation was of 0.97 ± 0.48 ms, while after 3 s in the presence of AE (0.1 mg ml^−1^), time in which more than 50% of *I*_Nav_ inhibition was regularly reached, this parameter was of 1.13 ± 0.7 ms. These values did not show a statistical difference.

**Figure 4:**
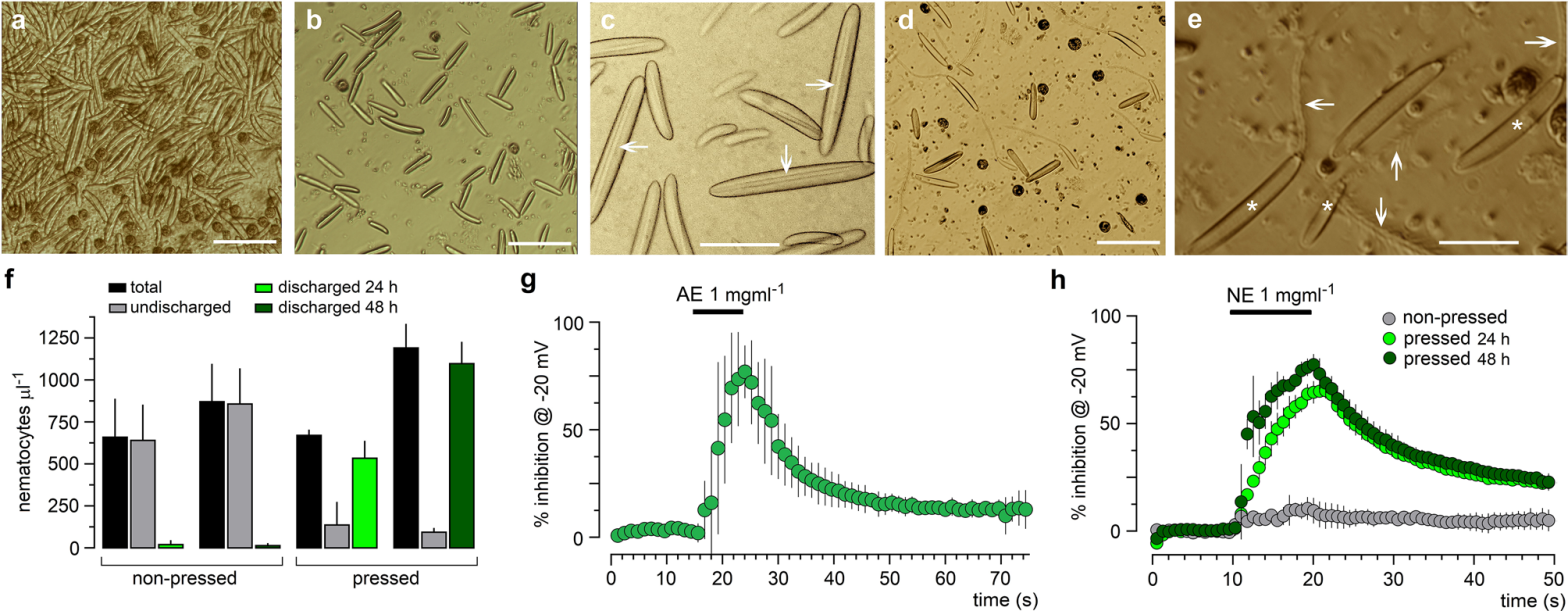
Nematocyst isolation and discharge using the French press cell disruption homogenizer. (**a**) Samples obtained from whole *B. annulata* anemone homogenate. (**b,c**) Samples recovered after 48 h following the Bloom’s method showing mainly undischarged nematocysts. Arrows in (**c**) signal typical images of twisted tubules in the undischarged nematocysts lumen. (**d,e**) Samples as in (**b**) after being processed through the French press at 6.2 × 10^7^ Pa, that provided mainly discharged nematocysts. Asterisks in (**e**) signal the nematocysts empty capsules and arrows indicate ejected tubules (bar = 50 μm in (**a,b,d**); bar = 20 μm in (**c,d**)). (**f**) Quantification of total (black columns), undischarged (gray columns), and discharged nematocysts (green columns) (per μl), for both non-pressed and pressed preparations in samples obtained by the Bloom’s method either after 24 h (light green) or 48 h (dark green) (mean ± S.D.; *p < 0.05 for comparisons between pressed vs. non-pressed). (**g**) Typical effect of AE (1 mg ml^−1^) on neuronal *I*_Nav_ response at − 20 mV (n = 6) using the same protocol as in Fig. 3. (**h**) A group of anemones from the same batch was processed by the Bloom’s method and pressed either after 24 or 48 h, and the corresponding extracts were assayed on the neuronal *I*_Nav_ activated at − 20 mV (light and dark green, respectively). In gray is shown the lack of effect of the non-pressed 48 h sample. Individual data points in (**g,h**) represent the mean ± S.D. from 4 to 5 neurons.

All the experiments described above used AE, the homogenate of *B. annulata*. Although it is known that this method successfully discharges nematocysts, the venom obtained with AE clearly may include substances from other anemone tissues in addition to the nematocysts content per se (e.g. Ref.^20^) To confirm that effects were elicited by nematocysts content, a procedure for nematocysts isolation was done followed by controlled discharge through mechanical means. Figure 4a shows the sea anemone homogenate visualized using phase contrast microscopy. This sample contained a large number of nematocysts, together with symbionts and the sea anemone tissue itself. Comparatively, using a modified Bloom’s method^42^, and settling periods of 24–48 h (Fig. 4b) provided with nematocysts free samples of most of other animal tissue, also significantly reduced the number of symbionts. An amplification of these preparations showed the typical morphology of loaded nematocysts containing twisted tubules in their lumen (arrows, Fig. 4c). The purified nematocysts samples obtained after 24–48 h were then pressed to 6.2 × 10^7^ Pascals using a French press. The extract obtained will be referred to as nematocysts extract (NE). The pressed preparations were then visualized under the microscope (Fig. 4d,e) to quantify the number of loaded and unloaded nematocysts after 24–48 h (Fig. 4f). Under the microscope, nematocyst discharge was visualized as an empty capsule associated with a large ejected tubule (asterisks and arrows, respectively in Fig. 4e). Figure 4f is a quantification made by triplicate of undischarged (gray bars) or discharged (green bars) nematocysts, in non-pressed aliquots compared with pressed aliquots from the same anemones samples. Quantification showed that in both 24-h and 48-h groups the efficiency of total nematocysts recovery increased, and that a small proportion of non-pressed nematocysts were already discharged. More importantly, it revealed that pressing led to a significant nematocysts discharge. For instance, from a total of 1190 ± 144 nematocysts per μl after 48 h of incubation, the pressed sample rendered 1096 ± 130 discharged nematocysts per μl; thus, most nematocysts were discharged after being pressed.

The time-course and potency of the effect for both AE and NE on neuronal *I*_Nav_, using the protocol as in Fig. 3d–f, is shown in Fig. 4g,h. First, 1 mg ml^−1^ AE inhibited *I*_Nav_ by 77.9 ± 12.6%, while NE (1 mg ml^−1^) obtained from the same anemones batch, incubated for 24 or 48 h, inhibited *I*_Nav_ by 56.8 ± 5.2% (light green circles) and 69.4 ± 4.4% (dark green circles), respectively (Fig. 4h). Both NE samples showed lower inhibition amplitude with a slower time-course compared to AE; however, the inhibitory effect was similar. The 48-h non-pressed (gray circles) sample presented low activity on *I*_Nav_ (peak inhibition of 9.2 ± 4.7%). Altogether, these data strongly suggested that the effect on *I*_Nav_ was due to a substance or substances contained in the nematocysts of *B. annulata*.

**Figure 5:**
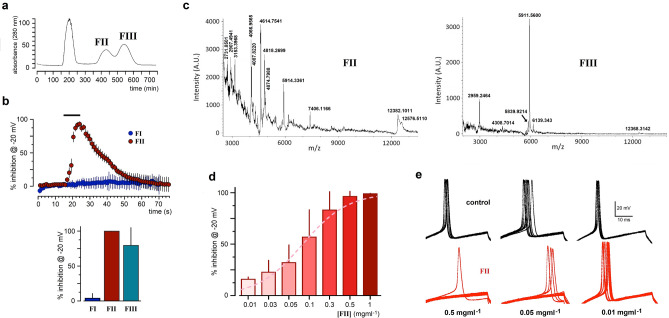
AE partial separation by Sephadex G-50 M chromatography, mass spectrometry, fraction dose-respond, and effect on the action potential. (**a**) Fractions obtained from AE gel filtration chromatography. Sephadex G-50 M column (90 × 8 cm) was equilibrated with PBS. Absorbance was monitored at 280 nm and fractions were collected at a flow rate of 300 μl min^−1^. Three absorbance peaks were detected and the corresponding fractions (FI, FII, and FIII) were collected and tested for effect on neuronal *I*_Nav_ monitored at − 20 mV (6–14 neurons in each case, held at − 100 mV and depolarized every 750 s for 20 ms). (**b**) The graph shows *I*_Nav_ inhibition time-course produced by FII (1 mg ml^−1^; 11 neurons), while FI did not produce a significant effect (4 neurons; mean ± S.D.), the column graph resumes the inhibition produced by each fraction (1 mg ml^−1^). (**c**) MALDI-TOF–MS spectrum obtained from the FII and FIII fractions obtained from AE gel filtration chromatography, the numbers indicated the mass for the main signals detected. In (**d**) FII was applied in distinct concentrations and a dose–response curve (in pink) was adjusted to data points indicating an EC_50_ of 80 ± 16 μg ml^−1^. (**e**) FII was tested on the neuronal action potential generated by depolarizing steps (35 ms, 0.75 nA) every 500 ms; three decreasing FII concentrations are illustrated. Traces in black (10 superimposed traces in each case) corresponded to control action potentials before FII superfusion, whereas red traces were obtained during FII application. 0.5 mg ml^−1^ potently inhibited the action potential, a concentration 10 times lower still blocked completely the action potential in few seconds, while 0.01 mg ml^−1^ FII was ineffective.

As a first approach for separation and purification of the toxins responsible for the effects, chromatography was performed with a Sephadex G-50 M column that allows substances separation based on their molecular weight. Chromatographic pattern from AE separated three peaks that were identified as FI, FII and FIII fractions (Fig. 5a). The bioassay of these fractions (Fig. 5b) on neuronal *I*_Nav_ indicated that 1 mg ml^−1^ FI did not affect *I*_Nav_, while FII was a potent fraction reaching inhibition values of 99.82 ± 0.47%. FIII also showed activity reaching an inhibition of 79.5 ± 25.9%, although its effect exhibited greater dispersion. The inhibitory time-course by FII was like that observed with AE and NE (Fig. 5b).

Since FII and FIII elicited *I*_Nav_ inhibition, a chemical analysis using MALDI-TOF mass spectrometry was done as this is shown in Fig. 5c. Both fractions showed peptides with molecular masses between 3 and 12 kDa, however, FII was more complex in composition than FIII. Importantly, the molecular masses observed were in the range of those reported for sea anemone neurotoxins (3–7 kDa)^6^. The presence of apparently equivalent components, in FII (e.g., m/z 5914.34 and 12,382.10) and FIII (m/z 5911.56 and 12,368.31) (Fig. 5c), might be an explanation for similar effects in both fractions.

The sensitivity of *I*_Nav_ to FII clearly presented a dose–response (D-R) relationship with a mean dose of 80.4 ± 16 μg ml^−1^ (Fig. 5d). Also, FII was used to analyze in cultured neurons its effect on action potential generation. For this, constant current pulses (0–65-0.85 nA) were applied to generate action potentials every 1–1.2 s at 30% above threshold amplitude, to avoid failures in impulse generation. Then, different solutions containing 10 μg to 1 mg of FII were applied for 10 s (n = 4, Fig. 5e). Complete action potential inhibition was observed in a dilution as low as 0.05 mg ml^−1^ after 3–4 s of superfusion, while a robust inhibition effectiveness was observed from 0.1 to 1 mg ml^−1^. FII produced a fast action potential blockage, and the duration was not affected during FII application. At all concentrations tested, the recovery of action potential amplitude was complete after washing.

**Figure 6:**
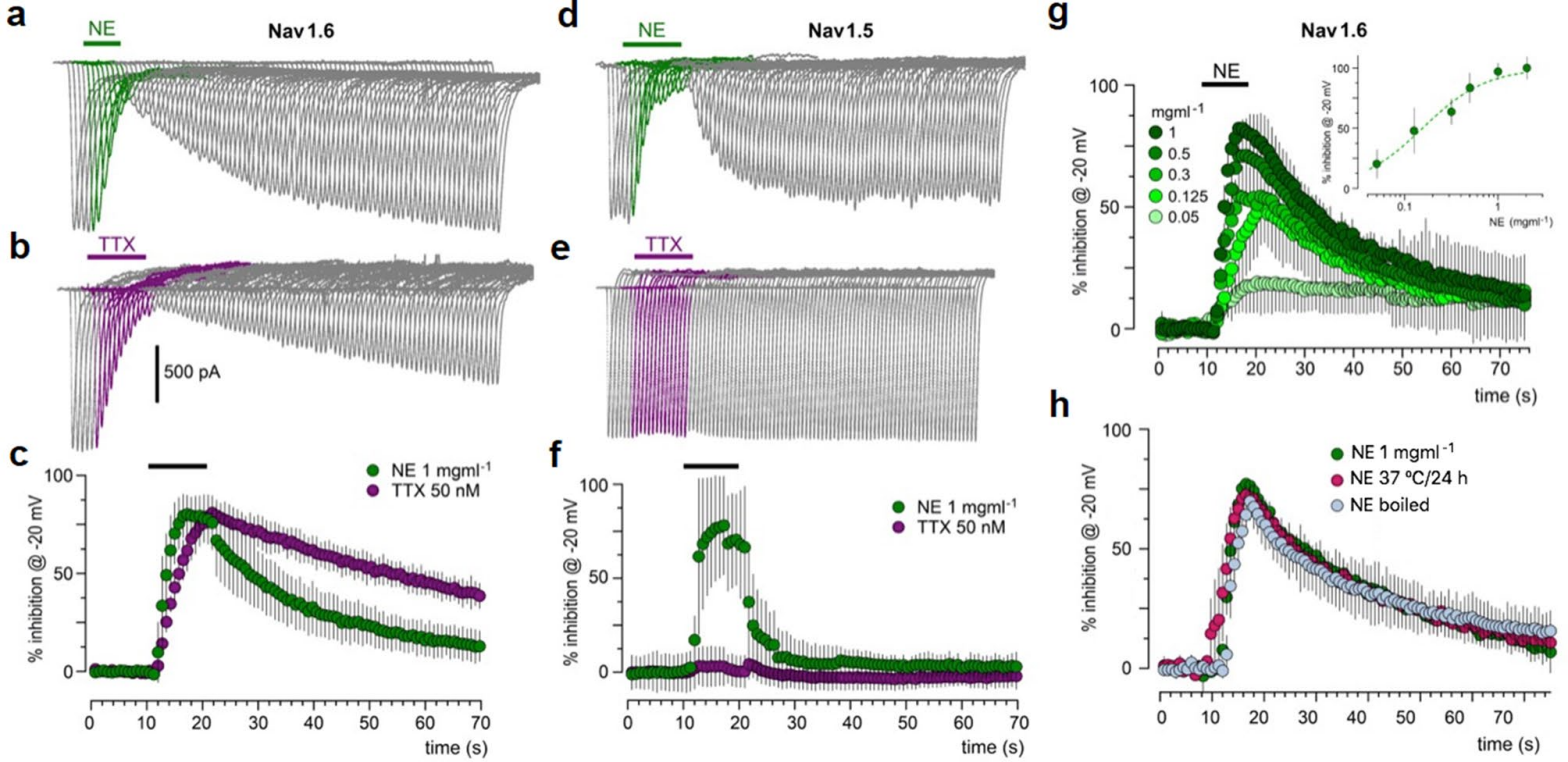
Nematocysts extract (NE) from *B. annulata* potently inhibited *I*_Nav_ elicited by opening of hNa_v_1.6 and hNa_v_1.5. (**a**) HEK293 cells expressing hNa_v_1.6 channels were recorded and *I*_Nav_ was activated applying voltage steps from − 100 to − 20 mV, using a similar protocol to that used in Fig. 3c. In the experiment illustrated, 1 mg ml^−1^ NE was applied as indicated by the bar in green after a control recording of *I*_Nav_ (traces in gray); during NE superfusion *I*_Nav_ decreased progressively (traces in green) and recovered during wash. (**b**) Traces show the effect of 50 nM TTX (purple traces) using the same protocol in cells from the same cultures. The graph in (**c**) shows the time-course of the inhibitory effect produced by each treatment (mean ± S.D. from 6 to 10 cells). (**d,e**) HEK293 cells expressing hNa_v_1.5 channels were recorded in similar manner and tested for either NE (traces in green) or TTX (traces in purple); as expected TTX did not affect *I*_Nav_, but NE strongly inhibited the response. The inhibition time-course produced by NE is illustrated in (**f**) together with the lack of effect by TTX; individual data points are the mean ± S.D. (5 cells in each case). (**g**) A dose–response relationship was built for the NE effect on the *I*_Nav_ in cells expressing hNa_v_1.6 channels (top graph); distinct NE concentrations from 1 to 0.05 mg ml^−1^ were applied to 6 different cells. Individual data points are the mean ± S.D. of the effect. Then, the normalized peak inhibition for each concentration was plotted (insert graph) and data points were fitted to a dose–response curve (green dashed line, EC_50_ = 155 ± 34 μg ml^−1^). (**h**) The graph shows the time-course of *I*_Nav_ inhibition produced by 1 mg ml^−1^ NE (green circles), and the effect produced by NE from the same batch that was boiled by 15 min (light blue circles) or NE samples that were incubated at 37 °C overnight (pink circles); individual data points are the mean ± S.D. from 5 cells in each case.

Tetrodotoxin (TTX) is a potent Na_v_ channel blocker^43^, the molecular identity of the TTX binding site on the channel is well known^44^. To know whether NE inhibition effect was produced acting on the same or similar TTX binding site, extract was tested in HEK293 cells expressing either hNa_v_1.6 or hNa_v_1.5 channels. As it is well known, TTX acts differentially on distinct *I*_Nav_ subtypes; hNa_v_1.6 is a TTX-sensitive channel, whereas hNa_v_1.5 is essentially a TTX-insensitive channel^45^. *I*_Nav_ currents in HEK cells were recorded similarly to those in neurons; thus, current responses were activated by depolarizing the cell periodically to values around the peak current. As is illustrated in Fig. 6a,c, 1 mg ml^−1^ NE superfusion inhibited the current elicited by hNa_v_1.6 opening as strongly as the blockage produced by 50 nM TTX (Fig. 6b,c). Remarkably, 1 mg ml^−1^ NE also strongly inhibited the current generated by hNa_v_1.5 channel activation (Fig. 6d,f). As expected, in these same cells the *I*_Nav_ current was not blocked by TTX (Fig. 6e,f). Also, comparing the rates of inhibition and recovery of NE and TTX on the hNa_v_1.6 current, NE seemed to act faster than TTX, and 155 ± 34 μg ml^−1^ NE inhibited 50% of *I*_Nav_ (Fig. 6g). Like that observed with AE, NE (1 mg ml^−1^) either boiled for 15 min or incubated for 24 h at 37 °C, fully retained activity (Fig. 6h).

### Generation of *I*_s_ response and GABA_A_ receptor activation

**Figure 7:**
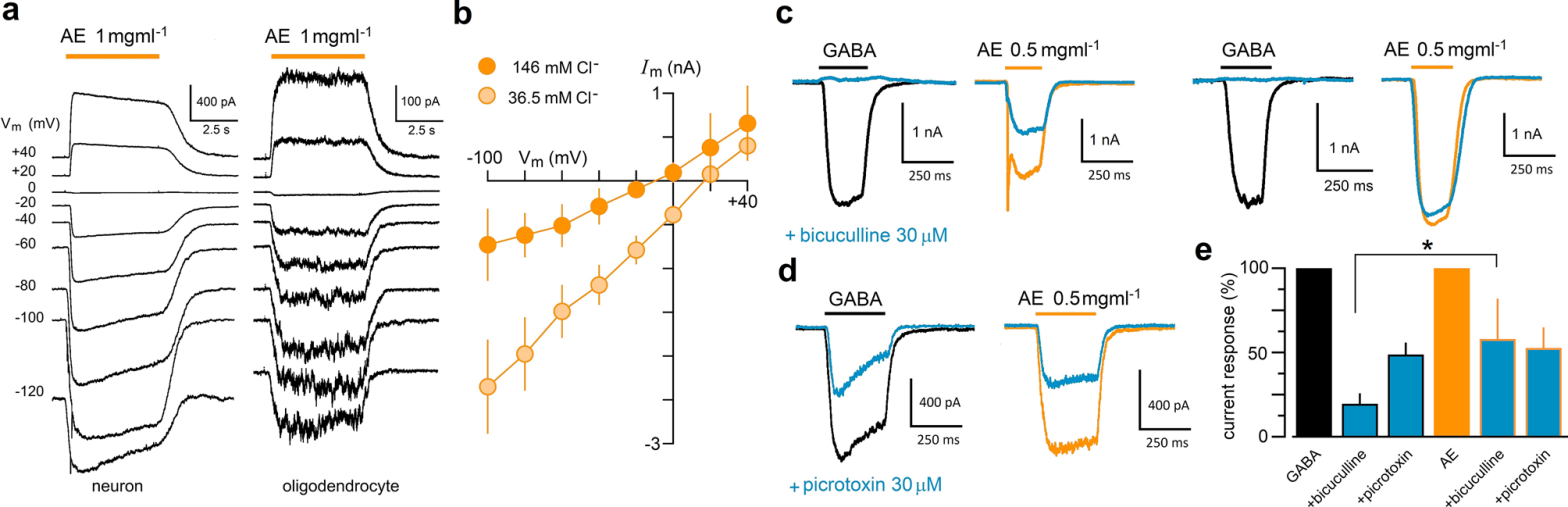
Ionic basis and pharmacology of inward currents generated by AE in neural cells. (**a**) Traces illustrate typical current response elicited by AE (1 mg ml^−1^) at different potentials either in neurons or in oligodendrocytes; in both cases extract generated inward currents that presented an E_rev_ close to 0 mV. (**b**) Current–voltage relationship for the response to AE were monitored in neurons in two conditions, first in control external solution containing 146 mM Cl^−^ (dark orange circles) and then the same cell in external solution containing 36.5 mM Cl^−^ (light orange circles). In low Cl^−^ external solution the E_rev_ shifted to more positive potentials. (**c**) Bicuculline (30 μM) was tested for its effect on the inward current response elicited by AE (0.5 mg ml^−1^). Neurons were tested for their response either to 5 μM GABA (black traces) or to AE (orange traces) and then the inhibitory effect of bicuculline (blue traces) was tested in each case. For GABA response bicuculline showed a strong inhibitory effect in all cases, while the effect on AE-elicited response was less potent and more variable between different neurons (two different neurons are illustrated). (**d**) In similar experiments, picrotoxin (30 μM, blue traces), inhibited in comparable manner to both the GABA- and the AE-response. (**e**) Normalized responses (mean ± S.D.) obtained in 8 neurons for each condition are shown in the bar graph. Current response in the presence of each drug was normalized against the corresponding amplitude reached by either GABA or AE applied alone (*p < 0.05 for comparisons between the GABA vs. AE amplitude values in the presence of bicuculline).

AE consistently generated a smooth inward current (*I*_s_) associated with an increase in membrane conductance in mRNA-injected oocytes (Fig. 2b–e). This response was generated in both neurons and oligodendrocytes, although the amplitude response was clearly weaker in the latter cell type, as illustrated in Fig. 7a. In oocytes, the estimated E_rev_ corresponded with the flux of Cl^−^; thus, the I–V relationship for the inward response was built for neurons (Fig. 7b). We also found that the E_rev_ for the inward current response in external solution containing 146 mM Cl^−^ was of − 8.8 ± 3.5 mV, while in solution with reduced Cl^−^ concentration (36.5 mM) the E_rev_ shifted to a potential of + 16.2 ± 4.3 mV, and the inward current amplitude became larger; altogether, these results suggest that inward current response was mainly carried by Cl^−^. The main Cl^−^ channel in neurons is GABA_A_R. To explore a possible GABA_A_R involvement in the response elicited by AE, the extract was coapplied in neurons with either 30 μM bicuculline or picrotoxin, an antagonist and a blocker of the GABA_A_R, respectively. In neurons (Fig. 7c–e; 5–7 neurons), bicuculline inhibited to 19 ± 18.1% the current elicited by 30 μM GABA, whereas picrotoxin blocked around 50% of the GABA response (to 48.2 ± 7.6%). In the same neurons, bicuculline and picrotoxin inhibited the current activated by 0.5 mg ml^−1^ AE to 57.5 ± 24.4% and 52.2 ± 12.8%, respectively (Fig. 7e). Contrary to what was observed in the GABA-generated current, bicuculline had a more variable effect on the inward current elicited by AE (see Fig. 7c), although picrotoxin presented a similar blocking effect either with GABA or AE stimulation (Fig. 7d).

The FI-FIII fractions obtained by chromatography also showed differential potency on neurons, thus 1 mg ml^−1^ FI was ineffective (7 neurons) to generate the inward response, 1 mg ml^−1^ FII generated inward responses of 1128 + 763 nA (13 cells), while FIII generated weak responses of 170.3 + 112 nA (7 cells), suggesting that the effect was elicited for a substance mainly contained in fraction FII.

**Figure 8:**
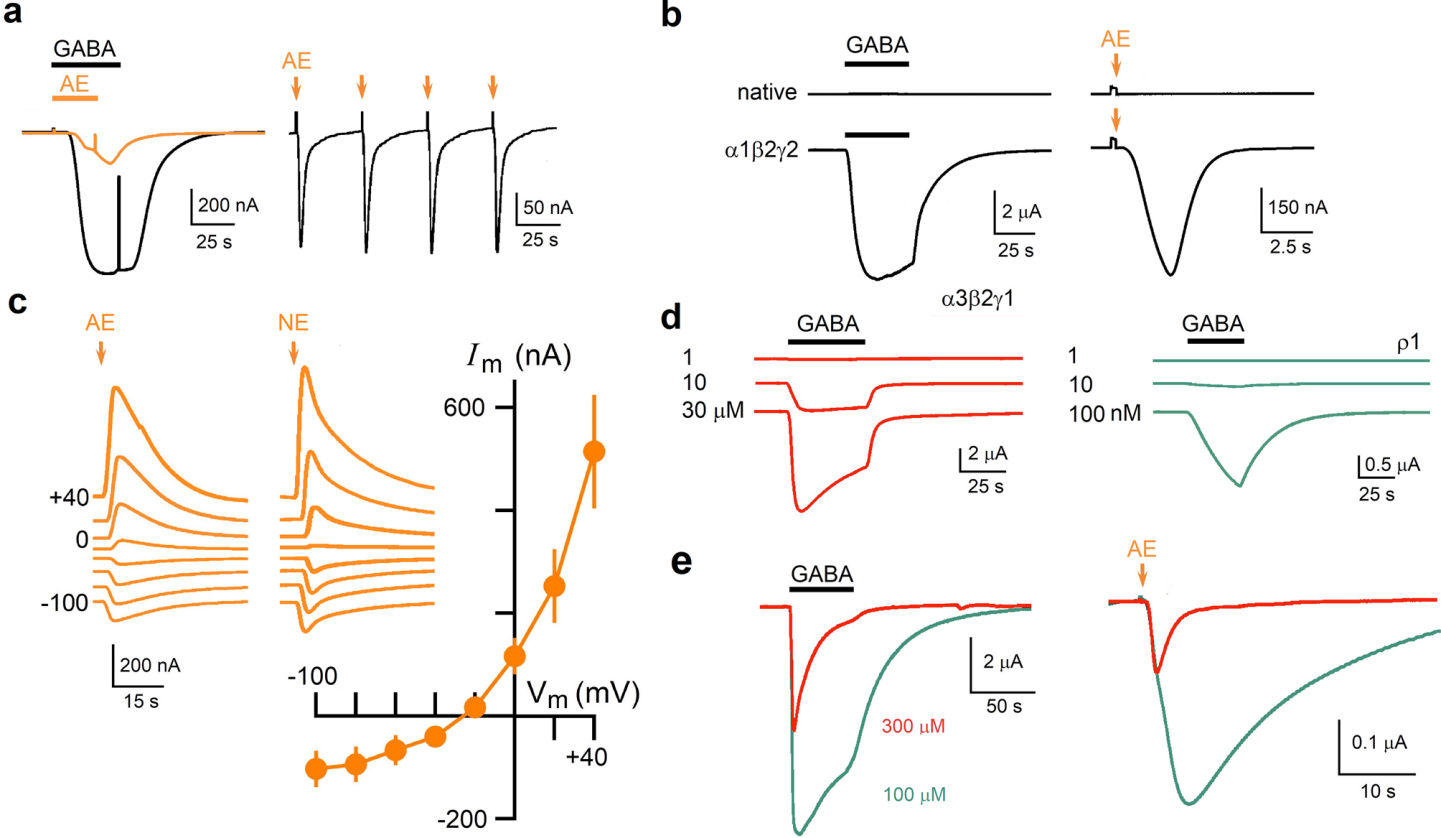
*B. annulata* venom contains an agonist of GABA_A_ receptors. (**a**) Traces on the left illustrate inward current responses in *Xenopus* oocytes expressing the neuronal GABA_A_ (α1β2γ2) receptor; cells were held at − 60 mV and superfused with either GABA (10 μM; black trace) or AE (1 mg ml^−1^; orange trace). In the trace on the right, AE was applied close to the oocyte surface using the jet delivery method, which consists of a small volume (25–75 nl) ejected from a micropipette filled with solution containing AE (0.5 mg ml^−1^), four AE-jets (orange arrows) applied successively show that current responses generated were robust and reproducible. (**b**) Top traces show that native oocytes (H_2_O-injected) were not sensitive to GABA superfusion or to AE jet-delivery (orange arrow indicates jet activation), while oocytes from the same frog expressing the neuronal GABA_A_ receptor (bottom traces) generated robust current responses to both GABA and AE. (**c**) Traces are responses at different holding potentials from − 100 to + 40 mV elicited either by jets of AE or NE as indicated; the graph is the current–voltage relationship of the responses by AE applying the same protocol in 6 different oocytes (2 frogs); individual data points are the mean ± S.D. of the peak current response in each potential. (**d,e**) AE (and NE) affects two more GABA_A_ receptor types expressed in oocytes, the oligodendroglial receptor α3β2γ1 (red traces) and the homomeric ρ1 receptor (green traces). Traces in (**d**) show the current response to GABA around the concentration threshold for each receptor, 1 μM for α3β2γ1 and 10 nM for the ρ1 receptor. In (**e**) the traces on the left illustrate responses elicited by a maximal concentration of GABA in oocytes expressing the α3β2γ1 receptor (in red) or the ρ1 receptor (trace in green), while the traces to the right show the response in the respective oocytes elicited by the same dose of AE. Traces are representative of results obtained in 7 oocytes from 3 different frogs.

To confirm a direct action on GABA_A_R by AE and NE, receptors were expressed in oocytes. Expression of the most common neuronal GABA_A_R, comprised by α1β2γ2 subunits, provides sensitivity to GABA superfusion (Fig. 8a). Oocytes responding to 10 μM GABA were also sensitive to 0.5 mg ml^−1^ AE superfusion, generating smooth inward currents that opened rapidly after beginning the application and stayed activated until GABA or AE were washed out. To make the application of AE more efficient, the extract was applied through a jet of solution ejected close to the oocyte surface. Extract solution jet application was a reproducible method of stimulation that consumed minimal amount of venom (Fig. 8a). The AE jet application method did not generate any response in native oocytes (Fig. 8b); however, in oocytes expressing the neuronal GABA_A_R, the delivery of either AE or NE prompted a rapid inward response that, as expected, had an E_rev_ of − 22.8 ± 2.6 mV (Fig. 8c). GABA_A_Rs composed of other subunits expressed in oocytes were also activated by AE and by NE applied in similar manner; thus, receptors comprised by α3β2γ1 subunits typically expressed in oligodendroglial cells^46^ and homomeric ρ1 GAB_A_R^47^ were also operated by the extracts. The α3β2γ1 receptor has an EC_50_ for GABA of 80 μM, with a threshold for the response close to 1 μM (Fig. 8d, red traces). The typical neuronal receptor expressed in the oocyte membrane has an EC_50_ around 100 μM with a threshold also close to 1 μM, while the homomeric GABA_A_R ρ1 has an EC_50_ of 1.2 μM, with a threshold within the nanomolar range (Fig. 8d, green traces). Using the same conditions and micropipette for jet application, responses to AE had an expected amplitude for the respective GABAAR expressed; thus, α3β2γ1-receptor responses were lower in amplitude, while ρ1-receptor responses were consistently larger (Fig. 8e).

These results suggested that extracts could contain GABA at a concentration within the micromolar range; thus, AE samples were analyzed using a GABA-specific ELISA test (with nanomolar detection sensitivity). However, AE and NE samples (3 different extracts) that activated robust responses in oocytes expressing either the α1β2γ2- or the α3β2γ1-receptor reported a GABA concentration of 9.97 ± 1.8 nM in the ELISA test, which by itself did not explain the observed amplitude responses. This strongly suggested that *B. annulata* venom contained a potent GABA_A_R agonist different to GABA.

## Discussion

More than sixty toxins affecting Na_v_ channels have been isolated from sea anemones, most of them cause a delay in Na_v_ channel inactivation^48,49^. The results described here show two novel toxic effects exerted by substances contained in the nematocysts of the sea anemone *B. annulata*, each one acting on fundamental membrane proteins of the mammalian nervous system: the Na_v_ channel and the GABA_A_R. The recognition of species of interest, due to specific effects of their toxins and/or by their venom potency, is undoubtedly an advantage to direct the search for new bioactive principles. Here we show that *B. annulata* venom contains substances that act on the nervous system of both vertebrates and invertebrates, as seen by the effects on crabs and on membrane proteins of vertebrates, specifically those from the nervous system of mammals (including human). The novelty of the effects described here, clearly indicates the importance of studying the potential bioactivity of cnidarian families that remain unexplored.

In several experiments of this study, nematocysts of *B. annulata* were first isolated using a modified Bloom’s method^42^, and then subsequently discharged in a controlled manner, applying pressure conditions that did not affect the symbiont structural integrity, as previously reported^50^. This increased the certainty that the effects observed were generated by compounds contained in the nematocysts, since extracts that were not pressed had reduced activity compatible with the basal discharge observed by nematocysts quantification.

Na_v_ and GABA_A_R channels are crucial in neural functions and effects on them could explain by themselves the venom lethality^30,51,52^. Na_v_ channels are responsible for nerve impulse generation, while GABA_A_Rs are important effector molecules of GABA, the main inhibitory neurotransmitter in the nervous system. Until now, most neurotoxins isolated from sea anemones (e.g., ShK, BgK, CgNa, and CGTX II) affect K_v_ or Na_v_ channels^13,53–59^. The peptide neurotoxins that act on Na_v_ channels are widely studied and have been classified into four types^6^. It is known that the action mechanism of many toxins from anemones involves their binding to open Na_v_ channels, causing a delay in channel inactivation and an increase in action potential duration^57,60,61^. However, different to this mechanism, our results showed that *B. annulata* venom contains a substance that inhibits the Na_v_ current response at the whole voltage range tested, without affecting both the inactivation rate, and apparently neither the voltage dependency of activation. Also, *I*_Nav_ inhibition more probably caused a rapid and dose-dependent blockage of the action potential generation in cortical neurons with no effect on its duration, suggesting that the inhibitory effect of *B. annulata* venom was more compatible with a blockage of the Na_v_ channel pore, although a complete definition of the inhibition mechanisms involved will require of further experiments. The chemical identity of the toxin(s) remains to be elucidated; however, it would correspond to a heat-stable molecule of more than 3000 Da. These features agree with the results of mass spectrometry analyses of FII and FIII that showed the presence of peptides in the expected range. Thermostability of anemone toxins has been reported in several cases and, this characteristic has been associated with the stability provided by the presence of disulfide bridges in its structure (e.g., Refs.^6,51,62^). It is also evident that its action mechanism was not identical to that of TTX, because *B. annulata* venom inhibited both the human Na_v_1.6 and Na_v_1.5 channel current responses, which clearly differ in their sensitivity to TTX^45,63^. It is still possible that *B. annulata* bioactive substance, described here, shares an overlapping binding site with TTX, and some small change in Nav1.5 is enough to interfere with TTX and not with that of *B. annulata*.

A different binding site for pore blockage in Na_v_ channels is that of the cone snail toxin GIIIA^64^, more experiments will be necessary to establish its possible participation in the *B. annulata* venom effect. The functional study of new molecules that affect Na_v_ channels in mammals remains limited. Hence, the discovery of new template molecules that could act in a more specific and differential manner is of general interest for the design and development of novel molecular tools (e.g. Ref.^65^). Furthermore, Na_v_ channels regulation are important not only for nervous system physiology but also because of their possible role in various diseases including cancer^66–69^.

A second molecule in the venom, acting as a GABAergic agonist, also represents a new action mechanism for sea anemone toxins, and its rapid action opening the channel in the millisecond range, as well as the complete recovery upon wash, resembles the action of the full agonist muscimol produced by the fungus *Amanita muscaria*^70^. Several natural substances act on GABA_A_R and many are drugs that have powerful effects on the nervous system (e.g., picrotoxine, β-carbolines). Also, peptides that inhibit GABA_A_Rs have been previously described in marine animal venoms in at least two species*, Laticauda semifasciata* and *Conus imperialis*^71^. In the case of *B. annulata* venom, the active substance operated three GABA_A_R types with different molecular identities (either neuronal or glial heteromeric receptors, as well as the ρ1 homomeric receptor); thus, this suggested that the venom contained concentrations in tens of the micromolar range of GABA. The expression of the GABAergic signaling system has been documented in cnidarians where it has been related to nematocyst discharge regulation, among other functions^72^. However, results of ELISA assays with sensitivity to nanomolar GABA concentration showed that the venom contained GABA in around 10 nM. This indicated that the molecule responsible for GABA_A_R activation was of a different nature since this concentration was too low to activate either the neuronal or the oligodendroglial receptor. Alternatively, the venom could contain a potent positive modulator that would increase the GABA_A_R sensitivity to GABA.

Nevertheless, our results also suggested that GABA_A_Rs were not the only receptors activated by the venom. Thus, testing the venom on cortical neurons revealed that currents produced in some cells, persisted even after its co-application with bicuculline, a potent competitive antagonist of the GABA site. This finding could also be explained by a different competition mechanism between bicuculline with the active substance as compared with GABA. On the other hand, the importance of a molecule in the venom that depresses the activity of the central nervous system seems to be clear, and it is striking that this mechanism apparently is not common among the venoms studied so far, since combining it with a powerful Na_v_ channel blocker molecule would result in a fast-acting bioweapon.

The advancement of robust biochemical and molecular techniques will provide detailed information about the identity of substances contained in the venom of many species that remain unexplored, including cnidarians. This study showed that the common Caribbean *B. annulata* is a source of novel neurotoxins that potently target essential proteins for the functioning of the mammalian central nervous system.

## Conclusion

The number of marine species that synthesize bioactive molecules studied is far from being exhausted, these molecules are widely diverse, and their molecular targets are of great interest in terms of basic mechanisms and for the development of new therapeutic and experimental tools. The use of heterologous expression models for the recognition of target molecules, such as the expression in *X. laevis* oocytes, represents a useful method to recognize relevant effects of the venoms. Here, oocytes expressing mammalian brain ion channels and membrane receptors revealed effects of the *B. annulata* venom on two of the most important proteins for the functioning of the nervous system, Na_v_ channels and GABA_A_Rs.

## Materials and methods

### Animal ethics declarations

All animal studies complied with the ARRIVE guidelines. All the animals were treated with the utmost care to minimize their suffering and were handled in accordance with the *Guide for the Care and Use of Laboratory Animals* by the USA National Institutes of Health, as well as the Local Guidelines on the Ethical Use of Animals for Experimentation at the Universidad Nacional Autónoma de México. *B. annulata* sample collection was carried out through permit no. PPF-066/20 from Dirección General de Ordenación Pesquera y Acuicultura, Quintana Roo, México.

### Sea anemone collection and venom extraction

Specimens of *Bartholomea annulata* were collected from the reef lagoon at Puerto Morelos, Quintana Roo, México (20° 50′ 45″ N 86° 52′ 08″ W; 20° 50′ 55″ N 86° 52′ 52″ W) by SCUBA diving (Fig. 1).

The venom extracts were prepared using two different methods, as follows: (1) *Sea anemone macerate to obtain* AE was carried out as described elsewhere^29^. Briefly, whole sea anemone bodies were homogenized in deionized water to perform the nematocysts discharge. Rupture of nematocysts was monitored by microscopy until most nematocysts were discharged. The extract obtained was centrifuged at 3.2 × 10^3^×*g* at 4 °C for 10 min, the supernatant was decanted, and the same procedure was applied one more time. Finally, the supernatant was dialyzed using a pore size limit of 3000 Da, lyophilized and frozen until it was used. (2) *Nematocyst’s isolation and discharge* to obtain NE. To avoid contamination of the extract with other cells or tissues, sea anemones were washed with filtered (0.45 µm) sea water to remove sand and small stones^57^, then they were exposed to six freeze–thaw cycles. Sea anemone bodies were immersed in two volumes of distilled water kept at 4 °C and stirring every 10 min for 2 h (modified from Ref.^42^). Samples were filtered with a 100-µm-mesh and the filtered material was allowed to settle for either 24 h or 48 h. After the settlement period the sediment was washed three times, first with phosphate buffer solution (PBS, containing in mM: 2.74 NaH_2_PO_4_, 7.2 dibasic Na_2_HPO_4_) plus 0.01% Triton, and then twice with PBS alone. The precipitate was visualized under a microscope to monitor the number of undischarged nematocysts. Then, discharge of these organelles was carried out applying a pressure of 6.2 × 10^7^ Pa using a French press cell disruption homogenizer (SLM-AMINCO, Urbana, IL, USA). It has been reported that symbionts are affected applying a higher pressure of 8.3 × 10^7^ Pa^50^. Finally, samples were centrifuged at 10^5^ g for 1 h at 4 °C, and the supernatant containing the venom was dialyzed using a pore size limit of 3000 Da, lyophilized, and kept at − 30 °C.

### Toxicity test

Both extracts were tested in *Ocypode quadrata* crabs, collected from the Puerto Morelos beach. Aliquots (50 μl) of either extract dissolved in PBS, or PBS alone as a control, were injected through the third walking leg^29,33^.

### Size exclusion chromatography

The AE was fractionated using Sephadex G-50 M. The column was equilibrated with PBS. Samples dissolved in the same buffer were eluted to a flow rate of 300 μl min^−1^, with monitoring at 280 nm, and 5-ml fractions were collected. The fractions were concentrated with reduced pressure (“rotary evaporator”), dialyzed using a pore size limit of 3000 Da, and lyophilized.

### Mass spectrometry

Fractions FII and FIII were chemically analyzed using MALDI-TOF mass spectrometry. 5 μl of a saturated solution of sinapinic acid (> 99.0% purity for MALDI-MS, Sigma, St. Louis, MO, USA) were added to 5 μg of lyophilized of each fraction. 1 μl of this solution was deposited onto the MALDI plate and allowed to dry at room temperature. The spectrum was recorded on linear positive mode on a mass spectrometer (Microflex Bruker Daltonics, Bremen, Germany) equipped with nitrogen laser λ = 337 nm and a 20 kV acceleration voltage.

### mRNA and cDNA

The poly(A)-mRNA was purified from adult Wistar rat brains; for this, mRNA was extracted from the whole brain using the guanidinium/phenol/chloroform method followed by oligo(dT)-cellulose chromatography^73^. The mRNA pool used here was purified from 19 different extractions from the same number of brains, each extraction was analyzed for its quality by electrophoresis and the ratio of absorbance 260/280 nm, as well as for its expression capacity of ion channels and membrane receptors into oocytes from different frogs. It was dissolved to 1 ng nl^−1^ in water and stored at − 80 °C until its use for expression using *Xenopus* oocytes.

The cDNA coding sequences for α1, α3, β2, γ1, and γ2 subunits of the GABA_A_R were obtained as previously reported^46^ from neural tissue, while ρ1 subunit coding sequence was donated by Dr. Ataúlfo Martínez-Torres^47^. All subunits were amplified, and each fragment obtained was cloned into pXENEX1 vector at the Ncol, BamHI, and NotI sites, then plasmids were linearized with the Hind III enzyme and used as templates for cRNA in vitro synthesis using the T7 mMESSAGE mMACHINE kit following the standard protocol (Ambion Invitrogen, Grand Island, NY, USA). cRNA was dissolved to 0.1 ng nl^−1^ in water and used for heterologous expression in *Xenopus laevis* oocytes.

### Heterologous expression in *Xenopus* oocytes and electrophysiology

Oocytes at stages V and VI^74^ were dissected from ovary lobules of *Xenopus laevis* and microinjected either with 50 nl of mRNA (1 ng nl^−1^) purified from rat brain or 50 nl of solution containing GABA_A_ cRNA (5 ng per oocyte)^75^. After 48 h, the injected oocytes were treated with collagenase (0.5 mg ml^−1^) at room temperature for 30 min in normal frog Ringer’s (NR) solution (containing in mM: 115 NaCl, 2 KCl, 1.8 CaCl_2_, 5 HEPES, pH 7.0)^15^. Finally, oocytes were kept at 16–18 °C in sterile normal Barth´s solution (containing in mM: 88 NaCl, 1 KCl, 2.4 NaHCO_3_, 0.33 Ca (NO_3_)_2_, 0.4 CaCl_2,_ 0.82 MgSO_4_, 5 HEPES, supplemented with 70 µg ml^−1^ gentamycin, pH 7.4) until they were used for electrical recordings.

Two or more days after injection, the membrane currents were recorded using the two- electrode voltage-clamp technique^38^. Microelectrodes (1 MΩ) were inserted into the oocyte and transmembrane currents were monitored using an Axon GenClamp 500 B (Molecular Devices, San Jose, CA, USA) amplifier. Signals were digitized and stored using an analog-to-digital convertor (Axon DigiData 1200; Molecular Devices) and specialized software (pClamp v9; Molecular Devices)^75^.

Unless otherwise stated, oocytes were held at − 60 mV and continuously superfused (10 ml min^−1^) with NR. The venom and distinct neurotransmitters (serotonin, 5 HT; γ-aminobutyric acid, GABA; acetylcholine; glutamic acid) were applied regularly by superfusion. To activate voltage-dependent ionic channels, mainly Na_v_ channels, a protocol of voltage steps was applied to build the current–voltage (I–V) relationships. Voltage-step protocols were applied while oocytes were held at − 100 mV and membrane potential was changed from − 80 to + 40 mV in steps of 20 mV for 250 ms, current responses were measured either at the steady-state or at the peak of the inward current, for *I*_s_ response or *I*_Nav_, respectively.

Injection of 500 pmol ethylene glycol-bis(β-aminoethylether)*N*,*N*,*N*′,*N*′,-tetraacetic acid (EGTA) into oocytes was made by pneumatic pressure ejection from micropipettes containing 200 mM EGTA solution (plus 5 mM HEPES, adjusted to pH 7.0 with KOH)^38^.

The method of drug application by superfusion regularly requires of a high sample volume (10 ml per min of application) for oocyte recording. Here, a much more efficient delivery system for the extracts was used (Fig. 8). This method consisted of applying the extract sample, either AE or NE (in NR solution), through jets of solution from a micropipette placed near the surface of the oocyte (approximately 100 μm), while maintaining the general flow of the external medium at a minimum level of 0.5 ml min^−1^; then the extract contained in the micropipette was ejected by applying small pulses of pressure through the opposite end. The pressure (regularly 10–20 PSI) was controlled through a manometer as well as by modifying the pulse duration. In this manner small volumes of test solution were applied. Regularly jets of few nanoliters (25–100 nl) were enough to activate current responses in oocytes expressing GABA_A_Rs.

### Electrophysiology and culture of neural cells and HEK293 transfected cells

Primary culture of neurons was obtained from E18 Wistar rat embryos according to previously described procedures^76^. Briefly, neurons derived from cortical lobes were resuspended in B27 neurobasal medium plus 10% Fetal Bovine Serum (FBS) and then seeded onto poly-l-ornithine-coated (30 µg ml^−1^) 24-well plates bearing 12-mm-diameter coverslips at 1 × 10^3^ cells per well. The medium was replaced by serum-free B27-supplemented Neurobasal medium 24 h later. The cultures were essentially free of astrocytes and microglia and were maintained at 37 °C and 5% CO_2_. Cultures were used at 7–12 days in vitro (DIV).

Primary cultures of oligodendrocytes derived from optic nerves of 12-day-old Sprague–Dawley rats were obtained as described previously^77,78^. Cells were seeded on 24-well plates bearing 12-mm-diameter coverslips coated with poly-d-lysine (10 mg ml^−1^) at a density of 10^4^ cells per well. Cells were maintained at 37 °C and 5% CO_2_ in Sato medium^70^ and recorded from 1 to 2 DIV.

Also, venom extracts were tested on HEK293 (human embryonic kidney) cells expressing human Na_v_ channels (hNa_v_ 1.5 or hNa_v_ 1.6), donated by Dr. Rita Restano Cassulini^79^. Cells were incubated in Dulbecco’s modified Eagle medium (DMEM) with 10% FBS and antibiotic G418 (400 µg ml^−1^), at 37 °C with 5% CO_2_.

Whole-cell patch-clamp recordings of either neural or HEK293 membrane ionic currents were performed with an Axon 700B amplifier (Molecular Devices). Cells were constantly perfused with normal external solution (NES, containing in mM: 135 NaCl, 5 KCl, 2 CaCl_2_, 1 MgCl_2_, 10 HEPES, and 5 glucose adjusted at pH 7.3 with NaOH). The pipette was filled with normal internal solution (NIS, containing in mM: 5 NaCl, 130 KCl, 1 CaCl_2_, 1.8 MgCl_2_, 10 HEPES, 10 EGTA, 0.2 Na-GTP, and 2 Mg-ATP at pH 7.3 (KOH). Data were digitized at 5 kHz and low-pass filtered at 0.5 kHz. Currents were recorded at distinct holding membrane potentials depending on the experiment as specified in each case, digitized, and stored for analysis using the analog-to-digital converter Digidata 1400 (Molecular Devices) and pClamp10 software (Molecular Devices). Current–voltage (I–V) relationships were regularly built by changing the membrane potential from − 100 to + 40 mV in 10-mV steps of 250 ms while cells were held at − 100 mV. In some instances, cells were held at a desired membrane potential while a test solution was superfused or applied using the jet method, and the peak currents generated were I/V plotted. Time-courses of the effect on *I*_Nav_ were followed in cells held at − 100 mV and then depolarizing steps (20 ms) eliciting maximal current (usually to − 20 mV or − 10 mV) were applied periodically every 0.75–1 s. Usually, the control current was monitored for 10 s and then a test solution was superfused for 5–10 s to record the effect on *I*_Nav,_ this was followed by a washing period with NES.

In one set of experiments the action potential was recorded in neurons under current-clamp, in these cases depolarizing current pulses (0.65–0.85 nA) of 35 ms were applied to generate action potentials every 500 ms, after a recording period control, different dilutions of FII were applied to test their effect on the nerve impulse.

Dose–response curves were fitted to the equation:

$${\text{I}}/{\text{I}}_{{\max }} = \left[ {\left( {{\text{A}}1 - {\text{A}}2} \right)/1 + \left( {\left[ {{\text{extract}}} \right]/{\text{EC}}_{{50}} } \right)^{{{\text{nH}}}} } \right] + {\text{ A}}2,$$

by the method of nonlinear least-squares fitting, where EC_50_ is the half-maximal effective concentration for the extract, nH is the slope factor (Hill coefficient), A1 and A2 are the initial and final normalized current (I) values, respectively, and [extract] is the concentration of either FII or NE.

### ELISA for GABA

GABA detection in both AE and NE was assayed using GABA ELISA Kit (Aviva Systems Biology, San Diego, CA, USA) according to the protocol instructions. Briefly, titrated standards and diluted samples were placed in a microtiter well-plate previously pre-coated with an anti-GABA antibody. GABA-Biotin complex was added to each well and the plate was incubated for 60 min. After incubation, the plate was washed. Avidin-HRP conjugate was added to each well and incubated for 45 min. Finally, 3,3′,5,5′-Tetramethylbenzidine (TMB) substrate was incubated for 30 min. Samples were read at 450 nm on an ELISA standard microplate reader.

### Statistical analysis

All data are expressed as mean ± S.D. of at least 4 cells, and in the case of oocytes, cells were obtained from 2 to 7 different frogs. The means of two groups were compared using a Student's t-test, or when appropriate, by analysis of variance followed by post-hoc comparisons of individual means using the Bonferroni correction. Statistical analysis was performed using GraphPad Prism software (version 6; La Jolla, CA). Differences were considered to be significant at p < 0.05.

## Supplementary Information

Supplementary Figure S1.
