# Supplementary material for: Sea anemone Bartholomea annulata venom inhibits voltage-gated Na+ channels and activates GABAA receptors from mammals
Source: Sci Rep. 2022 Mar 30;12:5352. doi: 10.1038/s41598-022-09339-x (PMC8967859; doi:10.1038/s41598-022-09339-x)
Supplement: Supplementary file 1 — Supplementary Figure S1. [file 41598_2022_9339_MOESM1_ESM.pdf]

Sea anemone *Bartholomea annulata* venom inhibits voltage-gated Na^+ channels and activates $GABA_A$ receptors from mammals

by

Antònia Colom-Casasnovas, Edith Garay, Abraham Cisneros-Mejorado, Manuel B. Aguilar, Fernando Lazcano-Pérez, Rogelio O. Arellano & Judith Sánchez-Rodríguez.

Supplementary Figure S1

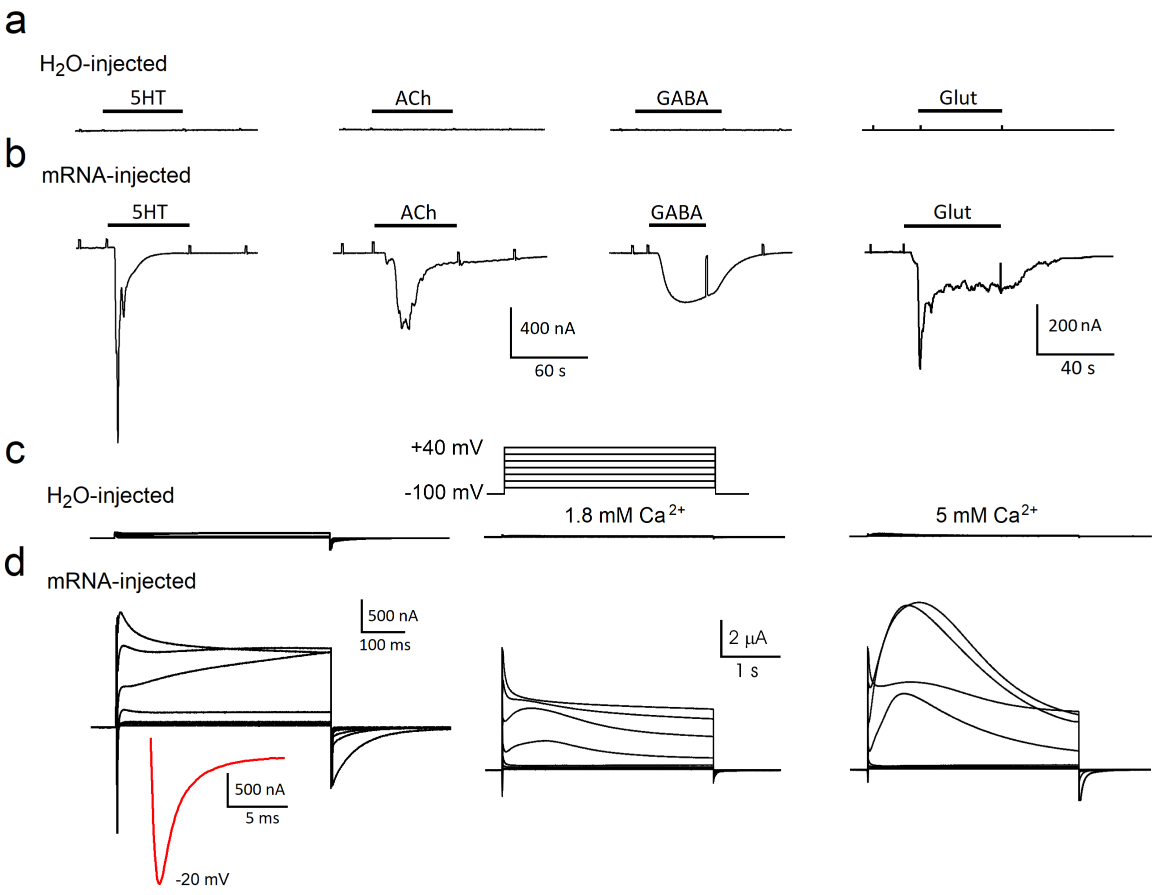

Figure S1. Electrical responses generated in control oocytes or in oocytes expressing mRNA from rat brain.

Frog oocytes expressing membrane proteins from the rat brain were used as a model system to screen effects produced by the venom from *B. annulata*. Series of traces in a)

and c) show current responses monitored electrophysiologically in control H_2O-injected oocytes, while traces in b) and d) were obtained in oocytes injected with and expressing mRNA from rat brain. In a) and b) oocytes were held at −60 mV and periodic (40–60 s) voltage steps to −40 mV (0.5 s) were applied to monitor membrane resistance; in both groups the responses elicited by several neurotransmitters (100 µM; applied as indicated by the top bar in each trace) were recorded. Robust current responses were observed in mRNA-injected oocytes but not in control oocytes and all neurotransmitters elicited inward currents associated with an increase in membrane conductance. In c) and d), oocytes were held at −100 mV and a voltage-step protocol was applied from -80 to +40 mV, with steps either of 250 ms or 5 s. Using the faster protocol, currents carried through Na^+ channels activation were better resolved in mRNA-injected oocytes (red trace shows in higher time resolution the I_{Nav} at -20 mV), while steps for 5 s show the T_{out} current responses that were activated superfusing NR solution or Ringer containing 5 mM $CaCl_2$. T_{out} currents corresponded with responses elicited by Ca^{2+} influx through voltage-dependent Ca^{2+} channels and opening of Ca^{2+}-dependent Cl^- channels. Voltage-dependent currents were well expressed in mRNA-injected oocytes but not in control cells.
